# Supplementary material for: Isolation of Chalcomoracin as a Potential α-Glycosidase Inhibitor from Mulberry Leaves and Its Binding Mechanism
Source: Molecules. 2022 Sep 6;27(18):5742. doi: 10.3390/molecules27185742 (PMC9504037; doi:10.3390/molecules27185742)
Supplement: Supplementary file 1 [file molecules-27-05742-s001.zip › molecules-1838875-supplementary.pdf]

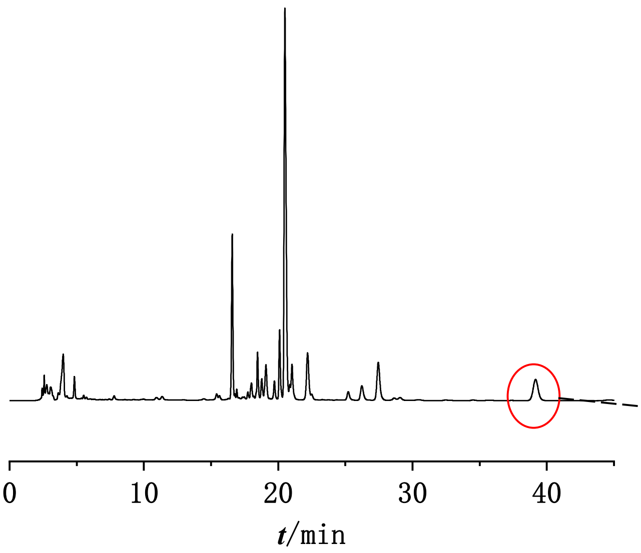

A. HPLC phase diagram before preparation

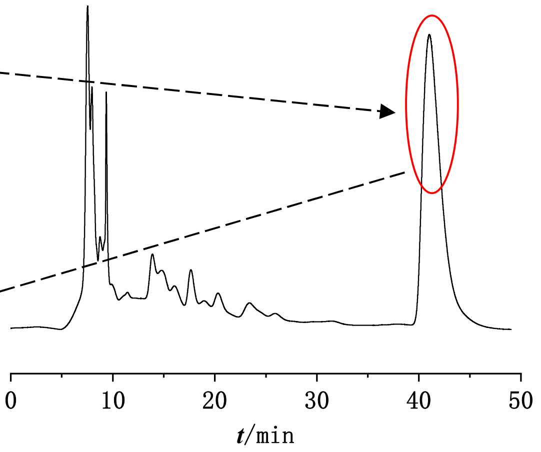

B. Preparative Liquid Diagram

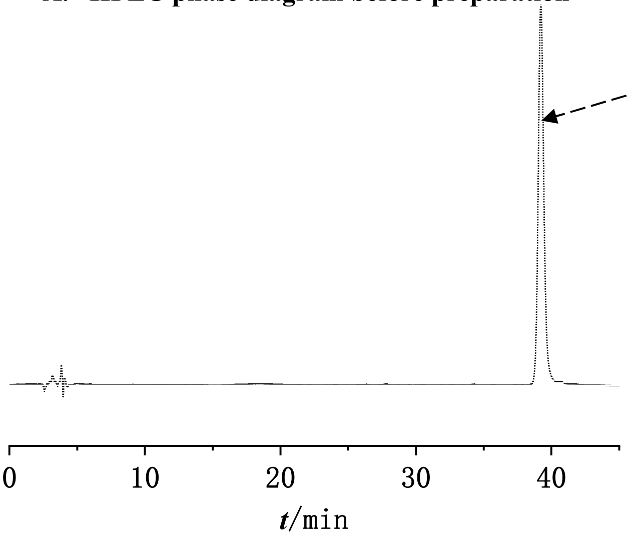

C. HPLC phase diagram after preparation

Figure S1. Liquid phase diagram during chalcomoracin preparation.

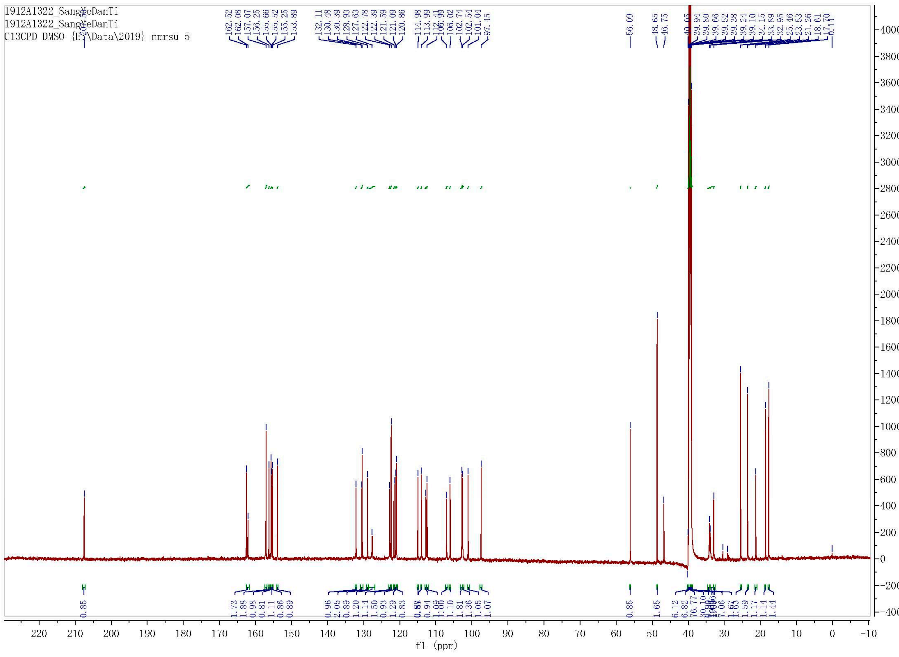

A.13C-NMR of chalcomoracin.

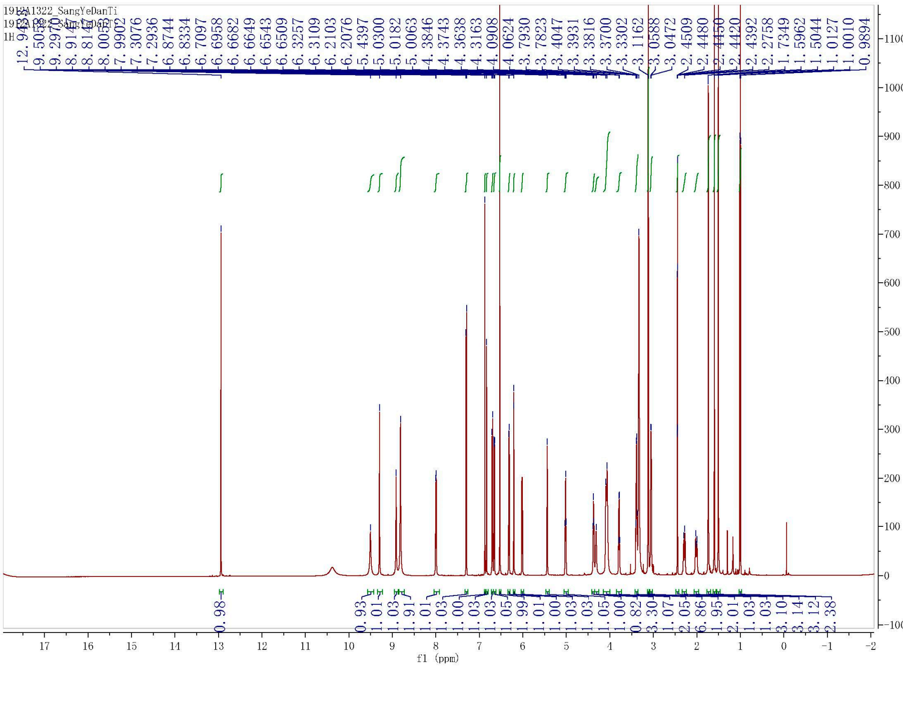

B. 1H-NMR of chalcomoracin.

Figure S2. NMR data of chalcomoracin.

Table S1. Experimental protocol for inhibitory activity assay.

Reagent	Blank (A1)	Control (A2)	Sample blank (A3)	Sample test (A4)
PBS buffer (7.4)	60 μL	30 μL	30 μL	0 μL
Inhibitor	0 μL	0 μL	30 μL	30 μL
α-Glucosidase	0 μL	30 μL	0 μL	30 μL
PNPG	50μL	50μL	50 μL	50 μL
Na_2CO_3	150 μL	150 μL	150 μL	150 μL

Table S2. The molecular interactions between chalcomoracin and α-glycosidase

Receptor		Interaction	Distance (Å)
VAL-544	N	H-acceptor	3.60
ASP-95	O	H-donor	3.43
ALA-93	O	H-donor	2.99
GLY-119	O	H-donor	2.76
ARG-275	NH1	H-acceptor	2.94
ARG-275	NH2	H-acceptor	3.32
ARG-275	NH2	Pi-cation	5.71
PRO-287	O	H-donor	3.03
